# Host innate immune responses and microbiome profile of neonatal calves challenged with *Cryptosporidium parvum* and the effect of bovine colostrum supplementation

**DOI:** 10.3389/fcimb.2023.1165312

**Published:** 2023-05-03

**Authors:** Lisa Gamsjäger, Karina M. Cirone, Steffany Schluessel, Mackenzie Campsall, Aydin Herik, Priyoshi Lahiri, Daniel Young, Antoine Dufour, Panagiotis Sapountzis, Saria Otani, Diego E. Gomez, M. Claire Windeyer, Eduardo R. Cobo

**Affiliations:** ^1^ Faculty of Veterinary Medicine, University of Calgary, Calgary, AB, Canada; ^2^ Laboratorio de Bacteriología, Grupo de Sanidad Animal, Unidad Integrada INTA, Universidad Nacional de Mar del Plata (UNMdP), Balcarce, Buenos Aires, Argentina; ^3^ Physiology and Pharmacology, Cumming School of Medicine, University of Calgary, Calgary, AB, Canada; ^4^ McCaig Institute for Bone and Joint Health, Cumming School of Medicine, University of Calgary, Calgary, AB, Canada; ^5^ Université Clermont Auvergne, Institut national de recherche pour l'agriculture, l'alimentation et l'environnement, Clermont-Ferrand, France; ^6^ National Food Institute, Technical University of Denmark, Kongens Lyngby, Denmark; ^7^ Department of Clinical Studies, Ontario Veterinary College, University of Guelph, Guelph, ON, Canada

**Keywords:** *Cryptosporidium parvum*, cattle, enterocolitis, colostrum administration, microbiota

## Abstract

**Introduction:**

Calves are highly susceptible to gastrointestinal infection with *Cryptosporidium parvum* (*C. parvum*), which can result in watery diarrhea and eventually death or impaired development. With little to no effective therapeutics, understanding the host’s microbiota and pathogen interaction at the mucosal immune system has been critical to identify and test novel control strategies.

**Methods:**

Herein, we used an experimental model of C. parvum challenge in neonatal calves to describe the clinical signs and histological and proteomic profiling of the mucosal innate immunity and microbiota shifts by metagenomics in the ileum and colon during cryptosporidiosis. Also, we investigated the impact of supplemental colostrum feeding on *C. parvum* infection.

**Results:**

We showed that *C. parvum* challenged calves experienced clinical signs including pyrexia and diarrhea 5 days post challenge. These calves showed ulcerative neutrophil ileitis with a proteomic signature driven by inflammatory effectors, including reactive oxygen species and myeloperoxidases. Colitis was also noticed with an aggravated mucin barrier depletion and incompletely filled goblet cells. The *C. parvum* challenged calves also displayed a pronounced dysbiosis with a high prevalence of *Clostridium* species (spp.) and number of exotoxins, adherence factors, and secretion systems related to *Clostridium* spp. and other enteropathogens, including *Campylobacter* spp., *Escherichia* sp., *Shigella* spp., and *Listeria* spp. Daily supplementation with a high-quality bovine colostrum product mitigated some of the clinical signs and modulated the gut immune response and concomitant microbiota to a pattern more similar to that of healthy unchallenged calves.

**Discussion:**

*C. parvum* infection in neonatal calves provoked severe diarrheic neutrophilic enterocolitis, perhaps augmented due to the lack of fully developed innate gut defenses. Colostrum supplementation showed limited effect mitigating diarrhea but demonstrated some clinical alleviation and specific modulatory influence on host gut immune responses and concomitant microbiota.

## Introduction

Neonatal calves are highly susceptible to *Cryptosporidium parvum* (*C. parvum*), a life-threatening apicomplexan protozoan parasite that causes watery diarrhea, dehydration, impaired gut absorption (Klein et al., 2008), and, in severe cases, impaired weight gain or death (Shaw et al., 2020). *C. parvum* is a zoonotic agent causing gastroenteritis in a diversity of vertebrates, including cattle, and it is a leading global cause of diarrhea, illness, and death in young children (Collaborators, 2017). Young children and immunocompromised individuals are at highest risk when in close contact with cattle, who are a main reservoir (Thomson et al., 2017). Cryptosporidiosis is a re-emerging food and waterborne disease for which currently there are no vaccines (Dumaine et al., 2020; Guo et al., 2022). Treatments usually rely on palliative oral electrolytes and a few labeled parasite-specific drugs (e.g., halofuginone) (Brainard et al., 2021), which have limited efficacy (Thomson et al., 2017; Riggs and Schaefer, 2020) and are legally restricted in some countries. Therefore, research has been focused on understanding cryptosporidiosis pathogenesis and gut-pathogen interactions, so that therapeutic targets or host anti-parasite immune mechanisms can be developed. However, the mucosal immune response during cryptosporidiosis in calves remains incompletely described due to a lack of consistent experimental models and limited tools to replicate the parasitic life cycle under laboratory conditions.

Bovine colostrum is essential for the protection of newborn calves. Its consumption within a short period of time after birth is critical for health (Chigerwe et al., 2015), long-term growth (Abuelo et al., 2021), and future productive performance (Armengol and Fraile, 2020). Oral administration of hyperimmune colostrum reduced shedding and clinical disease in lambs and calves (Perryman et al., 1999; Martín-Gómez et al., 2005; Askari et al., 2016). Such protective effects of colostrum have been largely attributed to the high concentration of pathogen-specific immunoglobulins G (IgGs); however, even non-hyperimmune colostrum has been shown to protect calves from undifferentiated diarrhea (Berge et al., 2009; Chamorro et al., 2017). This could be due to several important non-IgG biomolecules. Bovine colostrum contains epidermal growth factor (EGF) (Xiao et al., 2002) that sustains intestinal integrity (Clark et al., 2009) and inhibits *C. parvum*-induced intestinal disruption (Buret et al., 2003). Bovine colostrum is also abundant in lactoferrin (Stelwagen et al., 2009), lactoperoxidase, caseins, cathepsins, oligosaccharides (Arslan et al., 2021), and cathelicidins (Stelwagen et al., 2009; Isobe et al., 2013), which promote gut integrity in pigs (Otte et al., 2009; Yi et al., 2016). Other non-IgG biomolecules are non-coding RNAs and microRNAs (miRNAs) (Lau et al., 2001), present in the bovine mammary gland (Putz et al., 2019; Sun et al., 2019) and milk (Chen et al., 2010) in association with IgG (Ma et al., 2022), which are capable of epigenetically regulating gene expression of the host (Pritchard et al., 2012). However, research on bovine colostrum has been mostly focused on ensuring transfer of passive immunity and gut development in newborn calves, while its effect as a treatment at the onset of diarrheic cryptosporidiosis is unknown. This study aimed to determine the intestinal mucosal immune response and microbiome differences in calves experimentally inoculated with *C. parvum* and whether supplementation with a bovine colostrum product containing high levels of IgG would mitigate the disease.

## Materials and methods

### Sample cattle population

Male, Holstein, or crossbred Holstein x Ayrshire calves (*n* = 25) less than 1 day of age were acquired from pre-selected farms with no known history of diagnosed cryptosporidiosis. The number of calves per group (*n* = 6) represented a proof-of-concept experiment based on previous studies with successful *C. parvum* infection rates and development of colitis/diarrhea in challenged calves (Peeters et al., 1992). Only calves born by unassisted birth and without any overt signs of disease or congenital abnormalities were eligible for enrollment. Calves were transported within the first 12 hours of life to an isolation facility at the University of Calgary and, upon arrival, underwent a complete physical examination by a veterinarian or trained animal health technician. Only those deemed healthy were enrolled in the study. Within 4h of birth while still at the farm of origin, calves received one feeding of bovine colostrum (100 g of IgG, 1.4 liters; Calf’s Choice Total ^®^ powder, Saskatoon Colostrum Company Ltd, Saskatoon, Saskatchewan, Canada) by bottle, followed by an additional second (*n* = 25) and third (*n* = 21) similar feeding of the same bovine colostrum product within the first 24h of life either at the farm or upon arrival to the university facility. The third colostrum feeding was introduced due to low serum total protein concentrations observed in the first four calves. These four calves that received only 200 g IgG were evenly distributed among non-challenged treatment groups. Calves had *ad libitum* access to water and were housed individually in indoor pens that allowed visual contact among calves but no physical contact. Pens had rubber sleeping pads and wood shaving bedding, which was changed daily. Strict biosecurity protocols were employed to prevent the spread of pathogens among calves.

### Experimental groups

**Figure 1:**
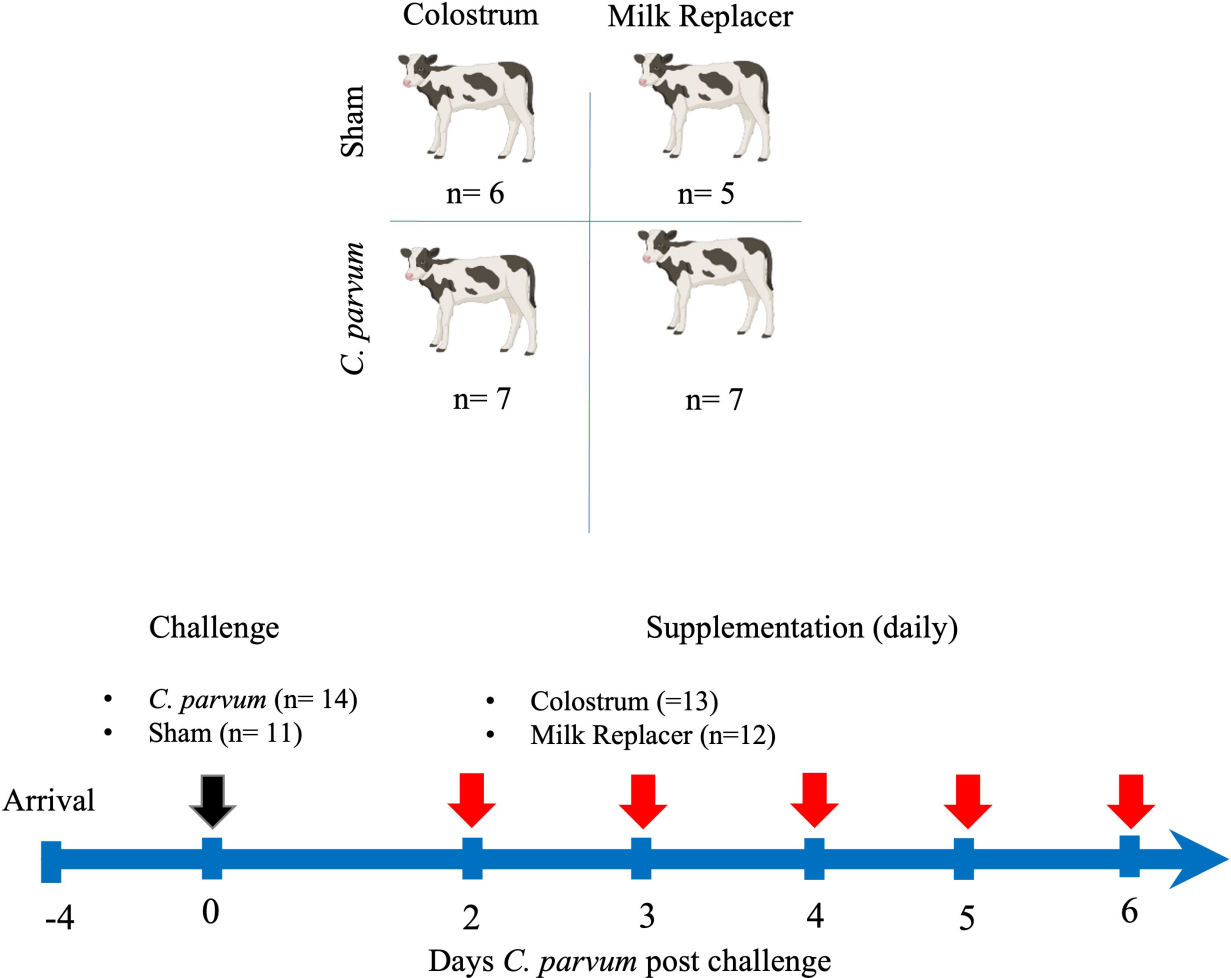
Experimental timeline. Schematic sequence of interventions, including groups of calves orally challenged (black arrow) with *C. parvum* (5 × 10^7^ oocysts) or inert solution (Sham) that were fed with milk replacer (MR) (*C. parvum*/MR; *n* = 7 and Sham/MR; *n* = 5) or supplemented with colostrum (C) (1 liter in the morning) (*C. parvum*/C; *n* = 7 and Sham/C; *n* = 6) from 2 to 6 days post-challenge (dpc). Feedings with milk replacer or colostrum are indicated by red arrows. Calves were euthanized at 6 dpc.

In a randomized controlled trial, calves were randomly allocated into one of four groups at 3–4 days of age: were randomly allocated into one of four groups: unchallenged (i.e., Sham) calves fed milk replacer (Sham/MR), unchallenged calves fed colostrum (Sham/C), *C. parvum* challenged calves fed milk replacer (*C. parvum*/MR), and *C. parvum* challenged calves fed colostrum (*C. parvum*/C). On the day of the experimental challenge (0-day post-challenge, dpc), calves in the *C. parvum*/MR *and C. parvum*/C groups were orally administered isolated peracetic acid-disinfected *C. parvum* oocysts (5 × 10^7^ Iowa isolate from Dr. MW. Riggs, School of Animal and Comparative Biomedical Sciences, University of Arizona) preserved in 5 ml of phosphate buffer solution (PBS) and mixed with 25 ml of milk (Riggs and Schaefer, 2020). Sham/MR and Sham/C calves received the same volume of inert PBS and milk. After the challenge, Sham/MR and *C.parvum*/MR calves were fed 2 liters of milk replacer (22% crude protein, 17% crude fat, and 0.15% crude fiber; Grober ProGro Milk Replacer, Grober Nutrition, Cambridge, ON, Canada) three times per day (8 a.m., 2 p.m., and 7 p.m.) *via* nipple bucket. Beginning at 2 dpc, Sham/C and *C. parvum*/C calves were fed bovine colostrum (1 liter, 100 g IgG/L, 60% crude protein, 19% crude fat; Saskatoon Colostrum Company Ltd, Saskatoon, SK, Canada) mixed with 1 liter of milk replacer in the morning feeding (8 a.m.) and 2 liters of milk replacer at the 2 p.m. and 7 p.m. feedings. If calves did not complete their feeding in the morning, the remaining volume of milk replacer or colostrum plus milk replacer was administered by tube feeder to eliminate any variability in colostrum consumption. The experimental design is depicted in **Figure 1**.

### Clinical examination and sampling of calves

Calves were examined daily, twice for Sham/MR and Sham/C calves and four times for *C. parvum*/MR and *C. parvum*/C calves, by trained individuals blinded to whether the calves were supplemented with colostrum or fed milk replacer only. Clinical parameters recorded included heart rate, respiratory rate, body temperature (normal: 38.5–39.5°C or abnormal: < 38.5 or ≥ 39.5°C), and a standardized health scoring system (Riggs and Schaefer, 2020) for attitude, hydration status, ability to rise, appetite, and fecal consistency with minor modifications (**Table 1**). Calves that reached an attitude, ability to rise, or hydration score of 2, or a fecal score of ≥ 3 received 50% of the subsequent meal as milk replacer and the other 50% as oral electrolyte solution (OES; V-lytes HE, Vetoquinol, Lavaltrie, Canada) (1:1) by nipple bottle or tube feeder. An attitude, ability to rise, or hydration score of 3, or an appetite score of 4 for two consecutive feedings was considered a humane end point, in which case the calf was euthanized by a veterinarian. The euthanasia protocol consisted of inducing anesthesia with xylazine and alfaxalone and then terminating calves using either potassium chloride (KCl) intravenously or a captive bolt gun.

**Table 1:** Standardized scoring system for clinical parameter assessment in calves.

	1	2	3	4
**Attitude**	Bright, alert, responsive	Somewhat depressed, dull	Depressed, minimally responsive	
**Hydration - Mucous membrane (MM) tackiness/color, capillary refill time (CRT), skin tent recovery (STR)**	Normal, CRT< 2 s, STR < 1 s, moist and pink MM	Mild, CRT 2-4 s, STR 2-5 s, slightly tacky MM	Severe, CRT > 4 s, STR > 5 s, very tacky MM	
**Ability to rise**	Eager to rise and stable	Willing to rise but needed assistance	Incapable of standing	
**Appetite**	Ravenous, aggressive	Normal	Decreased	Refused to suckle
**Fecal consistency**	Normal, solid	Slightly loose partially sunk into bedding	Loose, watery feces that predominantly sunk into bedding	Watery feces sprayed onto walls and sunk fully into bedding

As humane end points, any calf with an attitude, ability to rise, and/or hydration score = 3 and/or an appetite score = 4 for two consecutive feedings was humanely euthanized by an on-call veterinarian.

Fecal samples were collected and stored at −20°C for polymerase chain reaction (PCR) identification of *C. parvum* (0, 2, 4, and 6 dpc and on the first day of required OES treatment), screening for other enteropathogens including *Escherichia coli* (*E. coli*) K99, *Salmonella* sp., and bovine rota- and coronavirus (0 and 6 dpc) (Animal Health Centre, Abbotsford, BC, Canada), and microbiome profiling (0, 4, and 6 dpc). At 6 dpc, calves were terminated as explained above and, within 1h post-mortem, ileum, mesenteric lymph nodes, and proximal spiral colon and respective fluids were sampled.

### Gut and mucin barrier assessment

Colon and ileum samples (1 cm^2^) were washed twice in phosphate-buffered saline (PBS) and fixed in 10% neutral buffered formalin for 24h at room temperature. Tissues were dehydrated in increasing the concentrations of ethanol, embedded in paraffin wax, cut (5 μm), and mounted onto glass slides. Tissues were stained with hematoxylin (6765008; Thermo Fisher Scientific) and eosin (6766008; Thermo Fisher Scientific) (H&E) for histological examination and Alcian blue (Periodic acid–Schiff, PAS) for mucin layer assessment. For further study of goblet cell morphology and mucin barrier, lectin histochemistry specific against highly glycosylated proteins in mucin was conducted in formalin paraffined wax-embedded slides (Belote et al., 2018; Larzábal et al., 2020). Tissues were dewaxed and antigenic epitopes were heat-retrieved before incubating the slides with nuclei counterstain 4′,6-diamidino-2-phenylindole (DAPI) (Thermo Fisher Scientific, 1:1,000), and Alexa 647 conjugated wheat germ agglutinin (WGA) lectin (Thermo Fisher Scientific, 1:500 diluted in TBS buffer with bovine serum albumin (2% w/v); 20 min, room temperature). Slides stained with DAPI only served as control (Lindén et al., 2008). Slides were rinsed in distilled water, mounted with ProLong™ Gold Antifade Mountant (Thermo Fisher Scientific, Thermo Fisher Scientific, Waltham, MA, USA), and examined using a FluoView FV1000 confocal immunofluorescence microscope (Olympus Lifescience, Tokyo, Japan). Slides were examined, by individuals blinded to experimental group, for epithelial erosion, hemorrhage, leukocyte infiltration, and lectin blotted mucin barrier (Lindén et al., 2008; Erben et al., 2014) (**Table 2**). Images were taken with a ZEISS AXIO (ZEISS Innovation Center, Dublin, CA, USA) microscope (20X, NA 0.5) and analyzed by ZEN 2.6 (2018, Munich, Germany) software. Fluorescent WGA (green) was calculated using ImageJ 1.53c software (National Institute of Health, Bethesda, MD, USA) and represented as mean fluorescence intensity (MFI).

**Table 2:** Rubric for histological assessment of enterocolitis and mucin barrier in calves adapted from previous publications (Lindén et al., 2008; Erben et al., 2014).

Severity	Epithelial erosion	Hemorrhage/presence of erythrocytes	Leukocyte infiltration	Lectin blotted mucin barrier	Score
**Normal**	Intact epithelium	None	No infiltration	Homogenous	0
**Mild**	Mild erosion/loss of crypt architecture	Minimal	Minimal presence at epithelium	Goblet cell loss	1
**Moderate**	Moderate erosion/focal ulcerations	Moderate	Low levels presence in mucosal layer	Less number of GC and poorly filled with mucus	2
**Severe**	Severe erosion/extended ulcerations	Abundant	Abundant in mucosa	Absence of goblet cells	3

### Transcriptional expression of pro/anti−inflammatory cytokines and host defense peptides in ileum and colon

Relative messenger RNA (mRNA) concentration of cytokines *IL-8*, *IL-10*, *IFN-γ*, and *TNF-*α, and *cathelicidin 5* were determined in ileum and colon by quantitative real-time polymerase chain reaction (qPCR) using pre-designed primers (RT2 qPCR Primer Assay, Qiagen) specific for bovine (# GADPH PPB00298A, HPRT1 PPB00330A, TNFα PPB00153A, INFγ PPB00336A, IL10 PPB339A, and CATH5 PPB00921). Total cellular RNA was extracted from tissue homogenates (Thermo Fisher Scientific, Waltham, MA). Each sample was treated with chloroform and centrifuged (10 min, 12,000*g*, 4°C) and nucleic acids precipitated by isopropanol. Pellets were washed with ice-cold 70% ethanol and resuspended in RNAse-free water to obtain complementary DNA (cDNA) (Quantabio reverse transcriptase, Thermo Fisher Scientific, Waltham, MA). Target gene mRNA values were corrected relative to the normalizer, GAPDH. Data were analyzed using the 2^−ΔΔCT^ method (Bustin et al., 2009) and reported as mean fold change of target transcript levels in each experimental group.

### Proteomic profiling in ileum

Ileum samples from each calf were subjected to a quantitative shotgun proteomic analysis including high-performance liquid chromatography (HPLC) and mass spectrometry. Tissues were lysed in a buffer composed of 1% SDS, 200 mM HEPES (pH 8.0), 100 mM ammonium bicarbonate, 10 mM EDTA, and protease inhibitor cOmplete tablets (Roche, Mannheim, Germany). Disulfide bonds of 100 μg of total protein were reduced with 10 mM Tris (2-carboxyethyl) phosphine hydrochloride (Thermo Fisher Scientific, Waltham, MA) at 55°C for 1 h. The proteins were alkylated by incubation with 15 mM iodoacetamide (VWR) for 25 min in the dark at room temperature. Proteins were precipitated out of solution by adding 600 μl of ice-cold acetone and incubated at 20°C overnight. Samples were centrifuged at 8,000*g* for 10 min before resuspension in 100 μl of 50 mM triethyl ammonium bicarbonate. Proteins were trypsinized (Thermo Fisher Scientific, Waltham, MA) overnight at a 1:10 enzyme-to-substrate ratio. For tandem mass tag (TMT) 6-plex labeling (TMTsixplex™ isobaric label reagent set, Thermo Fisher Scientific, Waltham, MA), 0.8 mg of TMT reagent was resuspended in 41 μl of acetonitrile and samples were spun down quickly at 2,000 rpm (380*g*) for 10 s and incubated at room temperature for 1 h. A total of four biological replicates per group (*n* = 4 calves per group) were labeled with TMT reagents and one TMT tag (131) contained the pooled samples [i.e., equal amounts of peptides (20 µg) of each sample per group] and served as internal standards for normalizing the data across the groups. For labeling, peptides were incubated with TMT reagents (room temperature, 1 h), and the reaction was quenched by adding 8 μl of 5% hydroxylamine and incubated for 15 min at 25°C. Peptides with different labels were combined before 100% formic acid was added to each sample to reach a volumetric concentration of 1% formic acid. Samples were spun at 5,000 rpm (2,350*g*) for 10 min and desalted using Sep-Pak C18 columns (Waters, 130 mg WAT023501). Sep-Pak columns were conditioned with 1 × 3 ml 90% methanol/0.1% TFA, 1× 2 ml 0.1% formic acid. Each sample was loaded onto a column and washed with 1× 3 ml 0.1% TFA/5% methanol. Peptides were eluted off the column with 1 × 1 ml 50% ACN/0.1% formic acid and lyophilized. Peptides were resuspended in 1% formic acid and a BCA assay (Thermo Fisher Scientific, Waltham, MA) was used to determine the concentration of peptide in each sample. Samples were dried down and stored at −80°C.

Liquid chromatography and mass spectrometry experiments were performed on an Orbitrap Fusion Lumos Tribrid mass spectrometer (Thermo Fisher Scientific, Waltham, MA) operated with Xcalibur (version 4.0.21.10) and coupled to a Thermo Scientific Easy-nLC (nanoflow Liquid Chromatography) 1200 system. Tryptic peptides (2 μg) were loaded onto a C18 trap (75 μm × 2 cm; Acclaim PepMap 100, P/N 164946; Thermo Fisher Scientific, Waltham, MA) at a flow rate of 2 μl/min of solvent A (0.1% formic acid and 3% acetonitrile in LC mass spectrometry grade water). Peptides were eluted using a 120-min gradient from 5 to 40% (5 to 28% in 105 min followed by an increase to 40% B in 15 min) of solvent B (0.1% formic acid in 80% LC–mass spectrometry grade acetonitrile) at a flow rate of 0.3 μl/min and separated on a C18 analytical column (75 µm × 50 cm; PepMap RSLC C18; P/N ES803; Thermo Fisher Scientific, Waltham, MA). Peptides were electrosprayed using 2.3 kV voltage into the ion transfer tube (300°C) of the Orbitrap Lumos operating in positive mode. The Orbitrap first performed a full mass spectrometry scan at a resolution of 120,000 FWHM to detect the precursor ion having a mass-to-charge ratio (m/z) between 375 and 1,575 and with a range of +2 and +4 charges. The Orbitrap AGC (Auto Gain Control) and the maximum injection time were set at 4 × 10^5^ and 50 ms, respectively. The Orbitrap was operated using the top speed mode with a 3-s cycle time for precursor selection. The most intense precursor ions presenting a peptidic isotopic profile and having an intensity threshold of at least 2 × 10^4^ were isolated using the quadrupole (isolation window of m/z 0.7) and fragmented with HCD (38% collision energy) in the ion routing Multipole. The fragment ions (MS2) were analyzed in the Orbitrap at a resolution of 15,000. The AGC, the maximum injection time, and the first mass were set at 1 × 10^5^, 105 ms and 100, respectively. Dynamic exclusion was enabled for 45 s to avoid the acquisition of same precursor ions having a similar m/z ( ± 10 ppm).

For bioinformatic analysis, spectral data were matched to peptide sequences against a bovine and *C. parvum* UniProt protein database using the Andromeda algorithm (Cox et al., 2011) as implemented in the MaxQuant (Cox and Mann, 2008) software package v.1.6.0.1, at a peptide-spectrum match false discovery rate (FDR) of < 0.01. Search parameters included a mass tolerance of 20 p.p.m. for the parent ion, 0.5 Da for the fragment ion, carbamidomethylation of cysteine residues (+ 57.021464 Da), variable N-terminal modification by acetylation (+ 42.010565 Da), and variable methionine oxidation (+ 15.994915 Da). TMT 6-plex labels 126–131 were defined as labels for relative quantification. The cleavage site specificity was set to trypsin/P (search for free N terminus and for only lysine), with up to two missed cleavages allowed. An average of the normalized results for each group was calculated and followed by the ratio of each group comparison. The ratios were log _(2)_ transformed, and the significant outlier cutoff values were determined after log _(2)_ transformation by boxplot-and-whiskers analysis using the BoxPlotR tool.

### Shotgun metagenomic sequencing in feces

Fecal samples for each calf collected at 0 and 6 dpc were sequenced using a KAPA Dual-indexed PCR-free library in a 6000 NovaSeq. The raw reads were trimmed using Trimmomatic (Bolger et al., 2014) and filtered to remove adapters and Bowtie (Langmead et al., 2009) with the latest Bos Taurus RefSeq assembly (GCF_002263795.1) to remove cow reads. Filtered reads were mapped to the virulence factor database (mgc.ac.cn/VFs/) and a customized Metagenome Assembled Genomes database using KMA (Clausen et al., 2018). Data integration for pathway and Gene Ontology (GO) enrichment was performed with Metascape (Zhou et al., 2019) and STRING-db (Szklarczyk et al., 2019). For virulence feature characterization, read counts were transformed by converting counts to proportions, dividing gene counts by gene length, and correcting them by dividing by the total counts of VFdb features for each sample. Virulence gene counts were converted to abundance tables and imported in R-studio for further analysis. For microbiota characterization, MAG (Metagenome Assembled Genomes) catalogs (database) representative of the predicted bacterial genomes in the samples were used. The MAGs were built using MEGAHIT assembler (Li et al., 2015) and autometa (Miller et al., 2019) and filtered using CheckM to remove the ones below 85% completeness and > 5% contamination (Parks et al., 2015). The newly built MAGs and the ones from a previous study (Teseo et al., 2022) using dRep (Olm et al., 2017) were compared, thus creating a non-redundant dataset of representative genomes for the samples. For abundance matrices, the read counts per sample were estimated using KMA and corrected by converting counts to proportions [(MAG contig counts/contig length)/average genome size] calculated using MicrobeCensus (Nayfach and Pollard, 2015).

### Statistical analysis

Statistical analysis of clinical and histological data was performed with Prism (v9.0) (Graph Pad San Diego, CA, USA). The descriptive analysis was performed on all variables, and normality of the data was tested, when possible, by Kolmogorov–Smirnov test. Values were presented as mean and standard deviation (SD) or median and interquartile range (IQR), as appropriate based on the distribution. For consistency and comparisons among experimental groups, the average daily physical examination results were analyzed. Clinical parameters (i.e., heart rate, respiratory rate, temperature, hydration, attitude, appetite, fecal consistency, and ability to rise) were evaluated by repeated measures two-way analysis of variance (ANOVA), while histopathology scoring results were compared by one-way ANOVA. A Geisser–Greenhouse correction was applied when sphericity was not assumed due to the small sample size, followed by Tukey test for multiple comparisons. Clinical and histopathology scoring results are displayed as summary graphs of the means with bars representing SD or standard errors of the mean (SEM) as indicated in the figure legends. The number of OES treatments was compared by Chi-square or Fisher’s exact test, as appropriate.

For proteomic bioinformatic analysis, GO annotation, proteins basic functions, domain functional descriptions, and annotation of biological processes were studied by the UniProt-GOA database (http://www.ebi.ac.uk/GOA/), InterPro domain database (http://www.ebi.ac.uk/interpro/), and the Kyoto Encyclopedia of Genes and Genomes (KEGG) data- base (http://www.genome.jp/kaas-bin/kaas_main; http://www.kegg.jp/kegg/mapper.html). A two-tailed Fisher’s exact test was employed to test the enrichment of the differentially expressed proteins (DEPs) against all identified proteins. All the categories obtained after enrichment were collected and filtered for those categories that were at least enriched in one of the clusters with a *P*-value < 0.05. This filtered *P*-value matrix was transformed and clustered using one-way hierarchical clustering in Genesis. Clusters were visualized using the R Package pheatmap (https://cran.r-project.org/web/packages/cluster/). Pathway enrichment and protein networking were analyzed by an online meta-analysis tool (metascape.org) (Zhou et al., 2019). All DEPs’ database accession numbers or sequences were searched against STRING database (v11) for protein–protein interaction (PPI) analysis and interaction networks visualized in Cytoscape (Shannon et al., 2003). *P*-values of < 0.05 were considered statistically significant for all analyses [MaxQuant software (v.1.6.0.1) using a peptide FDR of 0.01].

For the metagenomic analysis, barplots were plotted using ggplot2 to visualize features overview. Differential representation analysis was performed using the indicspecies package. Alpha diversity was calculated using the absolute number of observed features (Sobs), and the Shannon index and groups were compared using the non-parametric Kruskal–Wallis tests. Beta-diversity ordination was calculated by either Bray–Curtis distances and non-metric dimensional scale (NMDS) or, alternatively, by imputing the 0s in the abundance matrices, CLR-transform them, and running principal component analysis. Significant contrasts were evaluated using the Adonis package in R.

## Results

### Clinical parameters and fecal shedding

**Figure 2:**
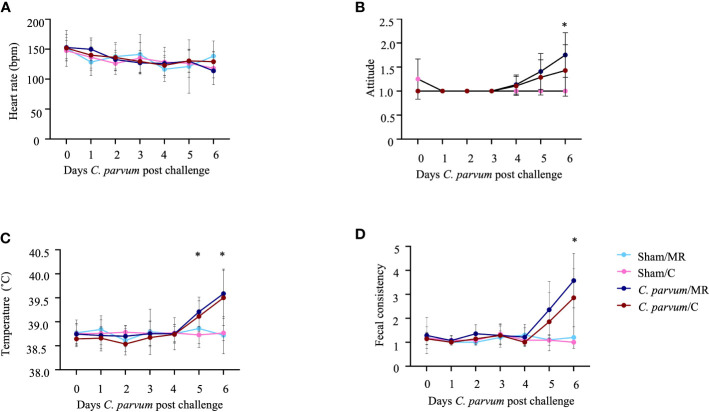
Clinical parameters in calves challenged with (*C. parvum* and supplemented with colostrum. **(A)** Heart rate. No significant differences among experimental groups. **(B)** Attitude score. Calves in the *C parvum*/MR group were significantly more depressed (*P* = 0.01) on 6 dpc when compared with Sham calves, whereas attitude scores of calves in the *C. parvum*/C group were not significantly different from Sham calves (*P* > 0.2). **(C)** Rectal temperature. Temperatures in the *C. parvum*/MR calves were significantly higher on 5 and 6 dpc (*P* = 0.02), compared with Sham calves, whereas there was no statistically significant difference between *C. parvum*/C calves and Sham calves (*P* = 0.07). Rectal temperatures of calves in the C.parvum/MR and C.parvum/C groups were similar 6 dpc (*P* = 0.9). **(D)** Fecal consistency. Average fecal scores on 5 and 6 dpc were significantly higher in *C. parvum*/MR and *C. parvum*/C calves (*P* < 0.005 and *P* < 0.05, respectively) compared with scores of calves in the Sham groups. Data are shown as mean ± SD. *P* < 0.05 (two-way ANOVA with *post-hoc* Tukey test for multiple comparisons) was considered significant.

A total of 25 calves (Sham/MR: *n* = 5; Sham/C: *n* = 6; *C. parvum*/MR: *n* = 7, *C. parvum*/C: *n* = 7) were enrolled in this study. The age at enrollment (i.e., day of sham inoculation or *C. parvum* inoculation) was not different for Sham/MR and Sham/C calves (mean = 3.6 days; SD = 1.1 days) compared with *C. parvum*/MR and *C. parvum*/C calves (mean = 4.4 days; SD = 1.3 days) calves (*P* = 0.5). Heart rates (range: 76 to 208 bpm; **Figure 2A**), respiratory rates (range: 20–88 bpm), appetite, hydration, and ability to rise slightly varied over time but did not differ substantially among experimental groups (*P* > 0.05). Calves in the *C. parvum*/MR group were more depressed (based on attitude score, *P* = 0.01) on 6 dpc when compared with Sham group calves, but calves in the *C. parvum*/C group were not significantly different from non-challenged groups (*P* > 0.2) (**Figure 2B**). Rectal temperatures were similar in all calves at enrollment (37.8–39.0°C) but increased in calves challenged with *C. parvum* (both *C. parvum*/MR and *C. parvum*/C) starting on 5 dpc compared with Sham calves (**Figure 2C**). Rectal temperatures in the *C. parvum*/MR calves were significantly higher on 5 and 6 dpc (*P* = 0.02; **Figure 2C**) when compared with Sham calves (Sham/MR and Sham/C), while there was no statistically significant difference among the *C.parvum*/C calves, Sham/MR, and Sham/C calves (*P* = 0.07). However, rectal temperatures of calves in the C.parvum/MR and C.parvum/C groups were similar 6 dpc (*P* = 0.9). Challenge with *C. parvum* provoked diarrhea as manifested by abnormal, loose to watery feces, regardless of colostrum supplementation in all but three calves. Average fecal scores on 5 and 6 dpc were significantly higher in *C. parvum*/MR and *C. parvum*/C calves (*P* < 0.005 and *P* < 0.05, respectively; **Figure 2D**) compared with scores of calves in the Sham groups.

None of the Sham calves met treatment criteria throughout the study period. Six and five of the seven calves in each of the *C. parvum*/MR and *C. parvum*/C groups required treatment with OES at least once, respectively. Calves in the *C. parvum*/MR group required significantly more OES treatments (*P* = 0.02) when compared with calves in the *C. parvum*/C group. Of the calves meeting treatment criteria, 10/12 were shedding *C. parvum* on the day of first treatment (*C. parvum*/MR: 6/6, *C. parvum*/C: 4/6). The first treatment occurred for one calf at 3 dpc (*n* = 1, *C. parvum*/C), for four calves at 4 dpc (*n* = 3, *C. parvum*/MR; *n* = 1, *C. parvum*/C), and six calves at 5 dpc (*n* = 3, *C. parvum*/MR; *n* = 3, *C. parvum*/C). From the total of 25 calves enrolled in this study, no calves had to be euthanized or excluded prior to the study end point at 6 dpc.

Regarding fecal shedding, no *C. parvum* DNA was detected on 2 dpc in *C. parvum* challenged groups but 4/7 C*. parvum*/MR calves (57%) and 1/7 (14%) *C. parvum*/C calves shed *C. parvum* at 4 dpc (*P* = 0.27, **Table 3**). At 6 dpc, the number of calves shedding *C. parvum* increased to 6/7 (86%) *C. parvum*/MR and 4/7 (57%) *C. parvum*/C calves (*P* = 0.56, **Table 3**). Both Sham/MR and Sham/C calves continued to have negative fecal *C. parvum* tests throughout the study.

**Table 3:** Genomic detection (+ , present; - , not present) of *C. parvum* in feces of calves orally challenged with *C. parvum* oocysts (0-day post-challenge, dpc), which were fed milk replacer (*C. parvum*/MR) or colostrum (*C. parvum*/C).

Days post-challenge (dpc)	*C. parvum*/MR (*n* = 7)							*C. parvum*/C (*n* = 7)						
	#1	#2	#3	#4	#5	#6	#7	#1	#2	#3	#4	#5	#6	#7
0	–	–	–	–	–	–	–	–	–	–	–	–	–	–
2	–	–	–	–	–	–	–	–	–	–	–	–	–	–
4	–	–	+	–	+	+	+	–	–	–	–	–	–	+
6	–	+	+	+	+	+	+	–	+	+	+	–	–	+

*Calves that received same volumes of PBS rather than *C. parvum* oocysts at 0 dpc and were fed milk (Sham/MR; n = 5) or colostrum (Sham/C; n = 6) were negative to *C. parvum* during the study.

### Ileal innate immune and pathological response and proteomic signature

**Figure 3:**
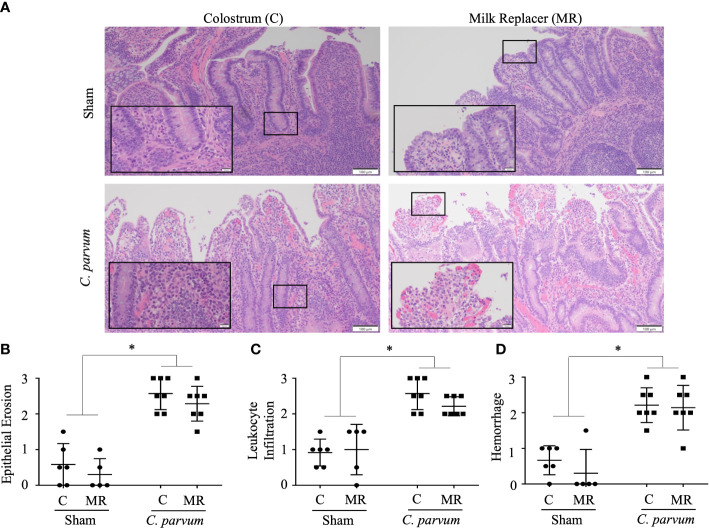
Grade of ileitis in newborn calves orally challenged by *C. parvum* and supplemented with milk replacer or colostrum. Sham and *C. parvum* (5 × 10^7^ oocysts) challenged calves were fed with colostrum (Sham/C and *C. parvum*/C, respectively) or milk replacer (Sham/MR and *C. parvum*/MR, respectively) and terminated at 6 dpc. **(A)** Representative microphotographs of ilea (H&E staining) taken with a ZEISS AXIO microscope (20X, NA 0.5) and analyzed by ZEN 2.6 (2018) software. Severity of ileitis was blindly scored by the grade of **(B)** epithelial erosion, **(C)** leukocyte infiltration in the lamina propria, and **(D)** hemorrhage following a scale represented in **Table 2**. Data are shown as mean ± SEM. *P* < 0.05 (one-way ANOVA post-hoc Bonferroni correction for multiple group comparison or two-tailed Student’s *t*-test for two groups) was considered significant.

*C. parvum* challenged calves (*C. parvum*/MR and *C. parvum*/C) showed histological hallmarks of ileitis accompanied by villous atrophy and necrosis that was not ameliorated by feeding colostrum (**Figure 3A** **)**. Ileitis in *C. parvum*/MR and *C. parvum*/C calves was manifested with severe grades of epithelial erosion, leukocyte infiltration in the lamina propria, and moderate mucosal hemorrhage (*P* < 0.05) (**Figures 3B–D** **)**. The expression of pro-inflammatory cytokines (*IL-8*, *IL-10*, *IFN-γ*, and *TNF-α* mRNA expression) and *cathelicidin 5* did not differ significantly in ilea of *C. parvum* challenged and unchallenged calves (**Supplementary Figure S1A**).

**Figure 4:**
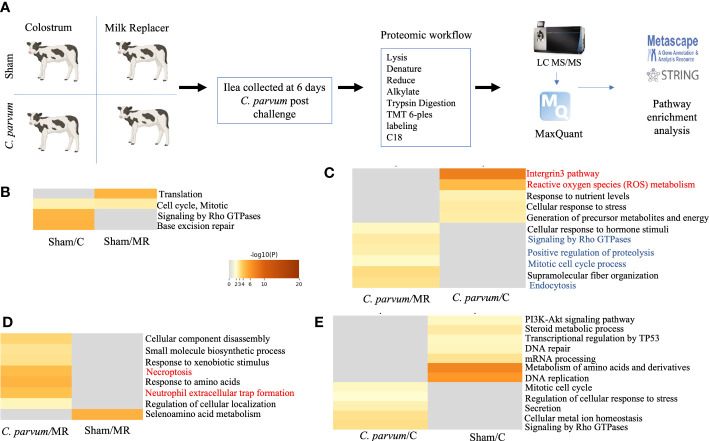
Proteomic immune-inflammatory signature in calves orally challenged by *C. parvum* and supplemented with milk replacer or colostrum. **(A)** Diagram of the proteomic workflow of different pathways in ilea from **(B)** Sham calves supplemented with colostrum or milk replacer (Sham/C *vs.* Sham/MR), **(C)** *C. parvum* challenged and supplemented with milk replacer or colostrum (*C. parvum/*MR *vs. C. parvum*/C, respectively), **(D)** *C. parvum* challenged or Sham supplemented with milk replacer *C. parvum*/MR *vs.* Sham/MR), and **(E)** *C. parvum* challenged or Sham supplemented with colostrum *C. parvum*/C *vs.* Sham/C). Ilea were collected at 6 dpc. Grade of red–yellow marks the level of upregulation.

Quantitative global shotgun proteomic followed by pathway enrichment using Metascape (metascape.org) of the ilea (**Figure 4A**) identified 17 unique proteins that were upregulated and 14 proteins that were downregulated in Sham/C compared with Sham/MR ilea (**Figure 4B** and **Supplementary Table S1** **).** Those proteins in Sham/C ilea were related with two upregulated pathways: Rho GTPases and base excision repair, and one pathway downregulated: translation (**Figure 4B** and **Supplementary Table S2** Sham/MR: Sham/C). Functional interactions between the DEPs as studied by STRING-db v11 (https://string-db.org) determined that four of the downregulated proteins in Sham/C (ATP-binding cassette subfamily F member 1 (ABCF1), glutamyl-prolyl-tRNA synthetase 1 (EPRS1), ribosomal protein L37a (RPL37A), and nuclear cap-binding protein subunit 1 (NCBP1), were clustered within a common translation pathway (**Supplementary Figure S2A**). Additionally, the upregulated expression of complement C1q-binding protein (C1QBP) showed to be related to both the upregulated and downregulated pathways in Sham/C ilea (**Supplementary Table S2** Sham/MR: Sham/C).

The proteomic expression in ilea of calves challenged by *C. parvum* and supplemented with either colostrum or milk replacer were compared. *C. parvum*/C compared with *C. parvum*/MR ilea displayed 30 upregulated and 24 downregulated proteins (**Figure 4C** and **Supplementary Table S1**). GO functional enrichment analysis indicated that integrin 3 and ROS metabolic process pathways, including proteins such as lipocalin 2 (LCN), were enriched in *C. parvum*/C ilea (**Figure 4C** and **Supplementary Table S2** *C. parvum*/C: *C. parvum*/MR). The downregulated pathways observed in *C. parvum*/C ilea included signaling by Rho GTPases, positive regulation of proteolysis, mitotic cell cycle process, and endocytosis (**Figure 4C** and **Supplementary Table S2** *C. parvum*/C: *C. parvum*/MR).

Given significant differences in the proteomic profiles in ilea, from cytoskeleton rearrangement pathways in Sham calves to ROS inflammation in *C. parvum* challenged calves, the contribution of colostrum supplementation to those local immune responses was investigated. In the case of milk replacer only, *C. parvum*/MR ilea compared with Sham/MR counterparts showed 16 upregulated and 15 downregulated unique proteins and two upregulated pathways: necroptosis and neutrophil extracellular trap formation (**Figure 4D** and **Supplementary Table S1**). A protein–protein interaction network was mapped showing proteins in *C. parvum*/MR compared with Sham/MR ilea that were upregulated including neutrophil extracellular trap formation pathway with MPO, rac family small GTPase 2 (RAC2), and IQ motif containing GTPase-activating protein 2 (IQGAP2) functionally clustered together (**Figure 4D**; **Supplementary Figure S2B**, and **Supplementary Table S2**). Another noticeable cluster of upregulated proteins in *C. parvum*/MR ilea was composed of TOP2A, DNMT1, SUPT16H, and HIST1H2AJ proteins involved in cellular component disassembly and necroptosis (**Figure 4D**; **Supplementary Figure S2B**, and **Supplementary Table S2** *C. parvum*/MR: Sham/MR). Among downregulated proteins in *C. parvum*/MR ilea, we found EPRS1, RPL23A, and RPS24, which clustered into seleno-acid metabolism pathways (**Figure 4D**; **Supplementary Figure S2C**, and **Supplementary Table S2**).

When colostrum supplementation was considered, *C. parvum*/C ilea showed 24 upregulated and 33 downregulated proteins compared with Sham/C, of which Rho GTPases and regulation of cellular response to stress pathways were upregulated in *C. parvum*/C ilea, but amino acid metabolism and DNA replication pathways were enriched in Sham/C ilea (**Figure 4E** and **Supplementary Table S2** *C. parvum*/C: Sham/C). When addressing proteins specific for *C. parvum*, four unique upregulated and two downregulated *C. parvum*-derived proteins were identified in *C. parvum*/C ilea compared with *C. parvum*/MR (**Supplementary Table S2** *C. parvum*/C: *C. parvum*/MR). These upregulated *C. parvum* proteins included proton-translocating NAD(P) ^(+)^ transhydrogenase and DNA helicase. Collectively, *C. parvum* induced grades of ileitis in young calves, whereas normal ilea expressed proteins related with cytoskeleton development and metabolism, ileitis provoked by *C. parvum* augmented proteins related with inflammation, neutrophil degranulation (ROS and MPO), and cellular necroptosis. The grade of ileitis was not ameliorated by colostrum supplementation or milk replacer. However, calves supplemented with colostrum sustained some proteins related with cytoskeleton dynamics compared with calves fed only milk replacer.

### Colon innate immune and inflammatory responses

**Figure 5:**
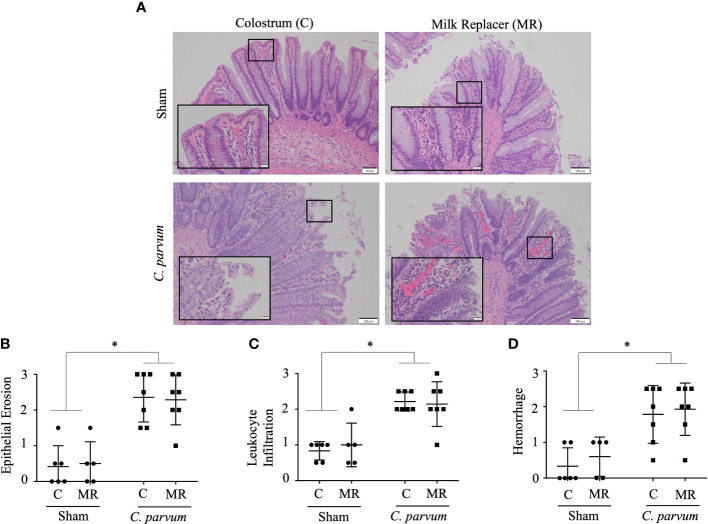
Grade of colitis in newborn calves orally challenged by *C. parvum* and supplemented with milk replacer or colostrum. Sham and *C. parvum* (5 × 10^7^ oocysts) challenged calves were fed with colostrum (Sham/C and *C. parvum*/C, respectively) or milk replacer (Sham/MR and *C. parvum*/MR, respectively) and terminated at 6 dpc. **(A)** Representative microphotographs of colons (H&E staining) taken with a ZEISS AXIO microscope (20X, NA 0.5) and analyzed by ZEN 2.6 (2018) software. Severity of colitis was blindly scored by the grade of **(B)** epithelial erosion, **(C)** leukocyte infiltration in the lamina propria, and **(D)** hemorrhage following a scale represented in **Table 2**. Data are shown as mean ± SEM. *P* < 0.05 (one-way ANOVA post-hoc Bonferroni correction for multiple group comparison or two-tailed Student’s t-test for two groups) was considered significant.

We next examined the colon, since this intestinal section can be colonized by *C. parvum* in severe immunosuppression or co-infections. For instance, while *C. parvum* typically infect the small intestine, *C. parvum* colonize the colons in gnobiotic pigs producing some degree of inflammation (Pereira et al., 2002). Likewise, *C. parvum* inhabit colons of neonatal mice co-infected with *Plesiomonas shigelloides* and *C. parvum* (Vitovec et al., 2001). We showed colons of calves challenged by *C. parvum* (*C. parvum*/MR and *C. parvum*/C) had areas of crypt hyperplasia along with dilated crypts and ulceration compared with Sham/C and Sham/MR colons, which displayed normal or minimally altered mucosa (**Figure 5A**). Colons from *C. parvum*/MR and *C. parvum*/C calves also showed similar severe epithelial erosion, infiltration of leukocytes in the lamina propria occasionally extending into the submucosa, and subepithelial extravasation of erythrocytes (**Figures 5B–D**). Transcription expression of *IL-8*, *IL-10*, *IFN-*γ, *TNF-α*, and *cathelicidin 5* genes, on the other hand, did not show differences between *C. parvum* challenged and unchallenged colons (**Supplementary Figure S1B**).

**Figure 6:**
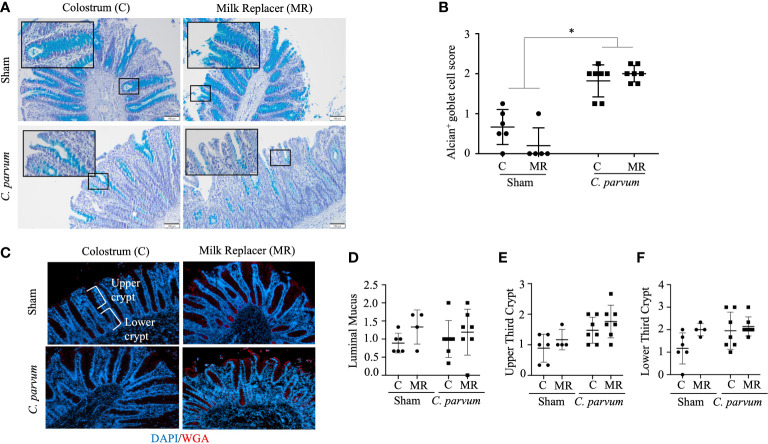
Mucin depletion in colons of calves orally challenged with *C. parvum* regardless of feeding regimen with milk replacer or colostrum. **(A)** Representative microphotographs of colonic goblet cells and **(B)** quantification of filled goblet cells per upper crypt (Alcian blue staining) in Sham and *C. parvum* (5 × 10^7^ oocysts) challenged calves fed with colostrum (Sham/C and *C. parvum*/C, respectively) or milk replacer (Sham/MR and *C. parvum*/MR, respectively) and terminated at 6 dpc. **(C)** Representative microphotographs of colonic mucus stained with WGA^+^ (specific for sialic acid and N-acetylglucosamine) lectins, counterstained with DAPI. Scoring of the lectin blotting for **(D)** WGA^+^ as 0 = absence in crypts; 1 = presence restricted to crypt base; 2 = throughout crypt; 3 = throughout crypt and in lumen. The photographs were taken using a 20× magnification. Data are shown as mean ± SEM. *P* < 0.05 (one-way ANOVA post-hoc Bonferroni correction for multiple group comparison or two-tailed Student’s t-test for two groups) was considered significant.

*C. parvum* migrates during colonization through the intestinal mucin barrier, a gel forming glycoprotein-rich layer composed mostly of mucins, including MUC2 (Li et al., 2021). Thus, the parasitic effects on the colonic mucin barrier were evaluated by the terminal oligosaccharide α-2,3 and α-2,6 N-acetylneuraminic acids, also known as N-acetylglucosamine (α-D-GlcNAc and NeuNAc; sialic acid) and mucin-producing goblet cells. *C. parvum* challenged colons, regardless of colostrum supplementation (*C. parvum*/MR and *C. parvum*/C), exhibited very few and incompletely filled goblet cells, which are typically restricted to the crypt bases (**Figure 6A**). In contrast, the mucin barrier in colons from Sham calves (Sham/C and Sham/MR) showed many goblet cells, although the mucin layer was still discontinued in several areas (**Figure 6A**). Scoring the mucin layer denoted equally marked alterations in the mucin barrier in *C. parvum*/MR and *C. parvum*/C colons compared with Sham/C and Sham/MR counterparts (**Figure 6B**). When mucin terminal sialic acid residues were assessed by specific WGA lectins, Sham/C and Sham/MR colons showed a continuous thin layer of WGA^+^ goblet cells homogenously distributed along the surface of crypts (**Figure 6C**). Fewer WGA^+^ goblet cells restricted to the crypt bottom and lesser mucin content were detected in *C. parvum*/MR and *C. parvum*/C colons (**Figure 6C**). The amount of luminal mucus and WGA^+^ goblet cell number along the crypt (upper and lower thirds) did not reach significant differences among groups (**Figures 6D–F**; *P* > 0.05).

### Intestinal pathobiome

Genomic material from *Bacillus* sp. (*n* = 3), haemolytic *E. coli* (*n* = 7), *Enterobacter cloacae* (*n* = 1), *Enterococcus* spp. (*n* = 10), *Klebsiella pneumoniae* (*n* = 7), *Lactobacillus* spp. (*n* = 11), and *Streptococcus* spp. (*n* = 9) was detected in feces from all calves (*n* = 25) regardless of whether calves were challenged with *C. parvum* or supplemented with colostrum (*P* > 0.05, **Table 4**). All calves were PCR negative for *E. coli* K99, *Salmonella* spp., and bovine rota- and coronavirus.

**Table 4:** Genomic presence (+ , present; - , not present) of aerobic bacteria in feces of Sham and *C. parvum* challenged calves (1- and 6-day post-challenge, dpc), which were fed with milk replacer (Sham/MR; *n* = 5; *C. parvum*/MR; *n* = 7) or colostrum (Sham/C; *n* = 6; *C. parvum*/C; *n* = 7).

ID	Group	*Bacillus* spp.		*E. coli* (haemolytic)		*E. coli* (non-haemolytic)		*Enterobacter cloacae*		*Enterococcus* spp.		*Klebsiella pneumoniae*		*Lactobacillus* spp.		*Streptococcus* spp.	
dpc		1	6	1	6	1	6	1	6	1	6	1	6	1	6	1	6
2	Sham/MR	–	+	–	+	+	+	–	–	+	+	–	–	–	–	+	–
4	Sham/MR	–	–	–	–	+	+	–	–	–	–	–	–	–	–	+	–
7	Sham/MR	–	+	–	+	+	+	–	–	–	+	+	–	–	+	–	–
8	Sham/MR	–	–	–	–	+	+	–	–	–	+	+	+	–	–	+	–
9	Sham/MR	–	–	–	–	+	+	–	–	–	–	–	+	–	–	–	–
1	Sham/C	+	–	–	–	+	+	–	–	+	–	–	–	–	–	–	+
3	Sham/C	+	–	–	–	+	+	–	–	+	–	–	+	–	–	–	+
5	Sham/C	–	–	–	–	+	+	–	–	+	–	–	–	–	–	–	–
6	Sham/C	–	+	–	+	+	+	+	–	+	–	–	–	–	+	–	–
10	Sham/C	–	–	–	–	+	+	–	–	–	+	–	+	–	–	–	–
11	Sham/C	–	–	–	–	+	+	–	–	–	+	–	–	–	–	–	+
12	*C. parvum/*MR	–	–	–	–	+	+	–	–	+	+	–	–	–	–	+	–
14	*C. parvum/*MR	–	–	+	–	+	+	–	–	–	–	+	–	+	–	–	–
15	*C. parvum/*MR	–	–	–	–	+	+	–	–	–	–	+	–	–	–	+	–
17	*C. parvum/*MR	+	–	–	–	+	+	–	–	–	–	–	–	+	–	–	–
19	*C. parvum/*MR	–	–	–	–	+	+	–	–	–	–	–	–	–	+	+	–
22	*C. parvum/*MR	–	–	–	–	+	+	–	–	+	–	–	–	–	+	–	–
24	*C. parvum/*MR	–	–	–	–	+	+	–	–	–	–	–	–	–	+	–	–
13	*C. parvum*/C	–	–	–	–	+	+	–	–	+	–	–	–	–	–	–	+
16	*C. parvum*/C	–	–	–	–	+	+	–	–	–	–	–	–	+	+	–	–
18	*C. parvum*/C	–	–	–	–	+	+	–	–	–	–	+	–	+	–	–	–
20	*C. parvum*/C	–	–	+	–	+	+	–	–	–	–	–	–	+	–	–	–
21	*C. parvum*/C	–	–	–	–	+	+	–	–	+	–	–	–	–	–	–	–
23	*C. parvum*/C	–	+	+	+	+	+	–	–	+	–	–	–	–	–	–	–
25	*C. parvum*/C	–	–	–	–	+	+	–	–	–	–	–	–	+	+	+	–

**Figure 7:**
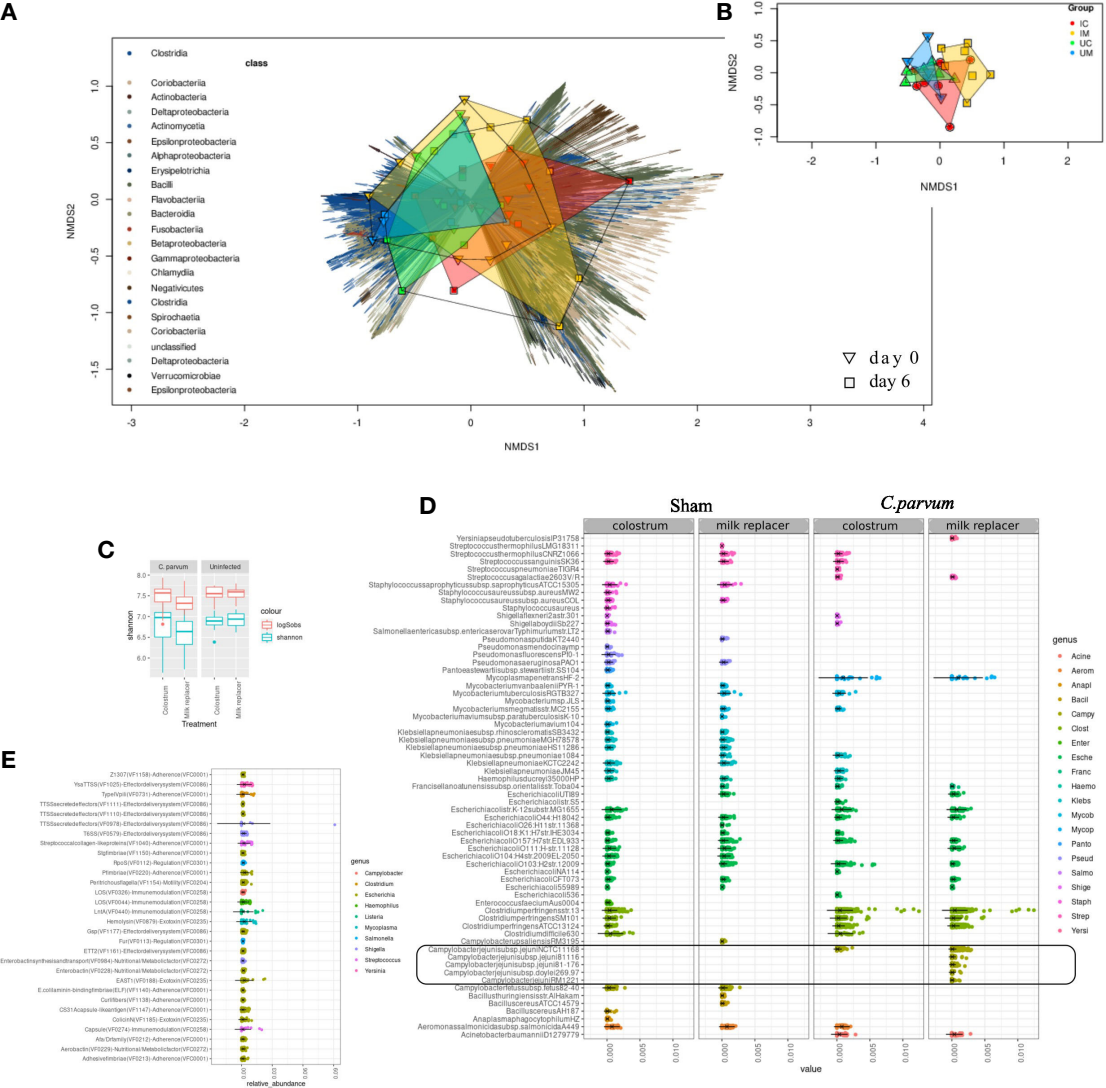
Distinct microbiome and pathobiome in newborn calves orally challenged by *C. parvum* and supplemented with milk replacer or colostrum. Microbiome **(A, B)** and pathobiome **(C–E)** changes were analyzed in feces from Sham and *C. parvum* (5 × 10^7^ oocysts) challenged calves fed with colostrum (Sham/C and *C. parvum*/C, respectively) or milk replacer (Sham/MR and *C. parvum*/MR, respectively). **(A)** Non-metric Multidimensional Scaling (NMDS) ordination of Bray–Curtis dissimilarity of all feces collected at 0 (reverse triangle) and 6 (squares) dpc. Dissimilarity abundance matrix represents composition based on the abundance of a reference MAG catalog built for this study (see Methods). Vectors indicate the bacterial class of each MAG contig and are color coded (left). Samples are color coded (top left) based on feeding regimen*infection status and shapes connect samples of the same group. **(B)** A similar NMDS ordination using only fecal samples from 6 dpc (*n* = 25). **(C)** Alpha diversity (light green) and richness (red) of virulence features in the four groups (Sham/C, *C. parvum*/C, Sham/MR, and *C. parvum*/MR). Dots represent means. **(D)** Overview of the most abundant (top 100) predicted virulence features in all fecal samples from the groups (Sham/C, *C. parvum*/C, Sham/MR, and *C. parvum*/MR) after excluding the genus *Aeromonas*. **(E)** A differential representation analysis across the groups (Sham/C, *C. parvum*/C, Sham/MR, and *C. parvum*/MR) at 6 dpc **(A–C)**. The features presented here are the ones that presented the sharpest contrasts between groups (indicspecies stat > 0.7, *P* < 0.05). Scatterplots show the abundance of the features in each sample. The rounded black square indicates the *Campylobacter* spp. features, which were exclusively present in *C. parvum*/MR calves.

By shotgun metagenomic sequencing assembled with previously reconstructed genomes (Teseo et al., 2022), and newly assembled MAGs with the data from this study, the entire microbial community and predicted virulence features were analyzed. From the metagenomic reads, 1,338 MAGs were mapped, and their abundance examined across fecal samples of 0 and 6 dpc. The alpha diversity differences were non-significant at 6 dpc (*C. parvum vs.* Sham *P* = 0.05; calf ID *P* = 0.157) (**Figure 7A**). In contrast, beta diversity ordination differed between *C. parvum* and Sham calf feces (**Figure 7A**) as demonstrated by different metrics (Bray Curtis *vs.* imputed, CLR and PCA; **Supplementary Figure S3**). Such beta diversity contrast was significant (*P* < 0.05), even though it explained only a small percent of the microbiome variation (*R* ^2 ^= 0.038) as most of it was driven by the natural variation between calves (*R* ^2^ = 0.611).

*C. parvum* challenged calves showed a microbiome distinct from Sham calves but challenged calves that received colostrum displayed a microbiome profile more comparable with the one observed in Sham calves (**Figure 7B**). Shannon alpha diversity, pondering the predicted virulence features, differed in feces from *C. parvum* challenged and Sham calves (fdr corrected *P* = 0.027), while a decreased richness was also noticed using Sobs index (fdr corrected *P* < 0.01) (**Figure 7C**). Virulence feature diversity was not driven by the animal (calf ID; *P* > 0.05). Virulence factor Type IV pili related to an adherence factor (VF0476) was abundantly expressed. However, because VF0476 is commonly found in *Aeromonas*, a ubiquitous bacterium in animals and the environment (Pessoa et al., 2022), further downstream analyses were done after excluding it. This refined analysis showed matches with exotoxins, adherence factors, and secretion systems related to *Campylobacter* spp., *Clostridium* spp., *Escherichia* sp., *Shigella* spp., and *Listeria* spp. in *C. parvum* challenged calves (**Figure 7D**). Analysis of virulence features adjusted to 6 dpc between *C. parvum* challenged and Sham calves (equal number of samples with and without *C. parvum* sequences) showed most of the identified potential virulence features were reduced when *C. parvum* was present but increased alongside *Clostridium* spp. counts (**Supplementary Figure S4**). These virulence features included components related to the Type IV pili and two system components of *Clostridium* spp. (**Supplementary Table S3**). The combined effect of colostrum supplementation and *C. parvum* at 6 dpc revealed a unique increase of *Campylobacter* spp. in *C. parvum*/MR (**Figure 7E**). Taken together, the challenge with *C. parvum* in newborn calves induced a drastic shift on overall microbiome and pathobiome defined by virulence factors, in part contributed by *Clostridium* spp. overgrowth that was even more pronounced in calves fed with milk replacer.

## Discussion

This study revealed gut innate immunological and microbiome signatures during cryptosporidiosis in neonatal calves and the effect of a daily regimen of colostrum supplementation on clinical and immune responses during the early establishment of *C. parvum*. The experimental model of *C. parvum* in neonates demonstrated that calves at this young age are extremely susceptible to cryptosporidiosis. Challenged calves experienced acute diarrhea and enterocolitis accompanied by depression and fever that required palliative oral treatment with electrolytes. Fever has been frequently reported in cryptosporidiosis, and we showed body temperature changed significantly in calves during the first week post-infection. The incubation time of 5 days for clinical signs agreed with data observed in 1-week-old calves experimentally challenged with *C. parvum* (Pohjola and Lindberg, 1986) and with the average 5–7 days observed in older calves (Abeywardena et al., 2015). Shedding of *C. parvum* after 4–6 dpc was expected based on other experimental studies reporting oocyte detection from 4 to 8 dpc (Pohjola and Lindberg, 1986). The high susceptibility to *C. parvum* in our calves resembles cryptosporidiosis in humans, where the disease is usually self-limiting in immunocompetent adult humans but is associated with severe diarrheal illness in children under 5 years old (Collaborators, 2017).

Colostrum has been proposed as therapeutic for unspecific diarrheas in pre-weaned calves (Carter et al., 2022), and colostrum administered with paromomycin in calves naturally infected with *C. parvum* impacted serum proteomes while reducing diarrhea and pathogen shedding in feces (Kacar et al., 2022). Our study deems that a prolonged colostrum supplementation instead of milk replacer, the usual diet for young calves in dairy production, did not categorically relieve diarrhea during the onset of cryptosporidiosis but did alleviate the concomitant fever and depression to some extent and was associated with lesser required OES treatments. This may potentially be attributed to the higher nutritional plane in calves supplemented with colostrum, which has previously been demonstrated to improve performance in calves infected with *C. parvum* (Ollivett et al., 2012). Of note, the limited sample size in this study could have precluded the finding of significant differences in fecal shedding among groups. Microscopically, challenged newborn calves, irrespective of feeding regimen, showed severe erosive and ulcerative ileitis, with accumulation of leukocytes in the lamina propria that extended to the colon. These lesions in colons of *C. parvum* challenged calves resembled those observed in murine ilea and colons inoculated with *C. parvum* oocysts (Al-Mathal and Alsalem, 2012), although those changes were not reported in previous experimentally challenged calves (Pohjola and Lindberg, 1986).

Because *C. parvum* oocyst encystation and colonization occur in the ileum (Al-Mathal and Alsalem, 2012), the local mucosal immunity during *C. parvum* infection in newborn calves was explored in this study. Alongside a leukocyte infiltration in the lamina propria of challenged calves, the proteomic data revealed *C. parvum* ileitis is characterized by an enriched ROS metabolic process pathway and the involvement of proteins associated with hypochlorous acid biosynthesis, lymphocyte aggregation, and thrombin-activated receptor signaling. Such mucosal alterations in the ilea of *C.parvum* challenged calves were not mitigated by colostrum supplementation. Neutrophil accumulation in the ileal mucosa alongside MPO activity was described in neonatal pigs mediated by the formation of superoxide (Zadrozny et al., 2006). The ilea of human patients with acquired immune deficiency syndrome (AIDS) also showed higher *C. parvum* infection with marked infiltration of neutrophils (Goodgame et al., 1995). However, the mucosal neutrophil influx would not be detrimental as neutrophils did not generate further peroxynitrite at mucosal surfaces and enhanced barrier integrity (Zadrozny et al., 2006). We also noted altered energy pathways in the ilea of *C. parvum* challenged calves in agreement with studies in the intestines of mice infected with *C. parvum* that showed depletion of glycolysis/citrate metabolite pathways (Karpe et al., 2021). Taken together, ileitis in *C. parvum* challenged neonates showed to course with active neutrophil inflammation and synthesis of ROS and other damaging inflammatory effectors.

Another consistent observation in *C. parvum* challenged calves was the depletion of the mucin barrier in the colon with extensive eroded surface areas, although sialic acid terminals were not particularly affected. The coating mucin conformed mostly by MUC2 secreted by goblet cells limits exposure to enteric pathogens (Bergstrom et al., 2010; Cobo et al., 2015; Cobo et al., 2017). Such lessened mucin barrier in challenged calves might have facilitated *C. parvum* colonization because bovine intestinal mucin and galactose-N-acetylgalactosamine (Gal/GalNAc) reduced *C. parvum* attachment to intestinal epithelia (Chen and LaRusso, 2000). Infection with *E. coli* O157:H7 in newborn calves also alters intestinal mucin barrier (Larzábal et al., 2020). Interestingly, even healthy control calves showed an impaired mucin layer in intestines, with fewer goblet cells restricted to crypt bottoms and thinner surfaces coated compared with adults. Butyrate and other short-chain fatty acids (SCFAs) contribute to mucus formation in the intestine during the first days of life (Liang et al., 2022), but butyrate-producing bacteria are not well established in the GI-tract of calves, as there is a transition from facultative anaerobes to anaerobic bacteria (Vlkova et al., 2006; Mayer et al., 2012). This could, at least in part, explain the difference in mucin between neonatal calves and slightly older calves, who show abundant mucin (Montagne et al., 2000a; Montagne et al., 2000b). Thus, the undeveloped mucin barrier in the gut this early in life infers a weakened constitutive defense with the conundrum of having to be settled during gut development but jeopardized by hyper mucin secretagogue effects of *C. parvum*. This would explain some of the susceptibility in neonates to cryptosporidiosis and other enteric diseases and highlights that dietary components could potentially promote intestinal mucin formation.

The general mechanisms of diarrheic enterocolitis implicate a disrupted intestinal epithelial integrity, mostly regulated by tight junction transmembrane proteins, that predisposes exaggerated amounts of flux escape, impaired water absorption, and microbial invasion (Walker et al., 2015; Liang et al., 2016). This study provides some evidence that *C. parvum* contributes to this “leaky gut syndrome” as ilea of healthy control calves displayed enriched proteins associated with actin and cytoskeleton dynamics, such as Rho GTPases and base excision repair pathways (Pradhan et al., 2021), that were lost during *C. parvum* infection. Disruptions in the intestinal architecture during *C. parvum* infection have been indeed observed in jejunums and ilea of mice which displayed upregulated actin, tubulin, and heat shock protein (Karpe et al., 2021). Additionally, *C. parvum* degrades occludin, claudin 4, and E-cadherin in murine enteroid-derived monolayer and intestines (Kumar et al., 2018). Pathogenic *E. coli* also disturbs genes associated with epithelial integrity in colons of newborn calves (He et al., 2022). Such overall rearrangements of the host cell cytoskeleton including aggregation and disassembly of actin would be key for the colonization of *C. parvum* (Yu et al., 2020) and other enteropathogens.

Enteric protozoa, including *C. parvum*, are known to promote shifts in microbial communities in infected animals. We showed *C. parvum* provoked a dysbiosis in neonatal calves, with higher occurrence of virulence genes attributed to exotoxins, adherence factors, and secretion systems from *Clostridium* spp. and other enteropathogens, including *Campylobacter* spp., *Escherichia* sp., *Shigella* spp., and *Listeria* spp. In agreement, calves naturally infected by *C. parvum* showed abundant *Clostridium perfringens* (Lucey et al., 2021), a bacterium associated with enterocolitis in suckling calves (Songer and Miskimmins, 2004). Additionally, the coexistence of *C. parvum* and *Campylobacter* spp. has been commonly reported in calves, and it would imply these enteropathogens synergize to rapidly colonize the gut of newborn calves and provoke diarrhea outbreaks (Grinberg et al., 2005). The documented co-occurrence of haemolytic *E. coli* (e.g., Shiga-like toxin producing *E. coli*) and *C. parvum* has been reported in calves associated with enteric infection outbreaks in children (Smith et al., 2004) and reinforce the role of cattle as reservoir of multiple enteric zoonotic pathogens. Cryptosporidiosis-induced dysbiosis has been previously reported in young mice (Mammeri et al., 2019) and calves (Ichikawa-Seki et al., 2019), which show an altered microbiota composition with increased *Bacteroidetes* and *Fusobacteria*, respectively. Similar gut dysbiosis was observed in C57BL/6J mice challenged by *C. parvum*, which displayed increased *Faecalibaculum*, *Barnesiella*, and *Lactobacillus* spp. in the small intestine and *Ruminococcaceae* in the caecum and colon (Karpe et al., 2021). This vulnerable microbiota shift in neonates seems to occur in other enteric diseases as well. A relative abundance of *Escherichia* sp. and *Shigella* spp. was noticed in colonic microbiota from newborn calves challenged by pathogenic *E. coli* (He et al., 2022) and in fecal microbiota of calves with naturally occurring diarrhea regardless of the pathogen (Gomez et al., 2017; Zeineldin et al., 2018; Jang et al., 2019; Gomez et al., 2022). Interestingly, we observed that microbiota from *C. parvum* challenged calves supplemented with colostrum could be more resilient compared with microbiota from calves fed only milk replacer. Colostrum ingestion is key immediately after birth, ensuring protective *Bifidobacterium* spp. and reducing opportunistic pathogenic *E. coli* and *Shigella* spp. in colons (Song et al., 2019). In addition, our finding supports that colostrum feeding may serve as a dietary intervention to regulate gut microbiota and improve gut health in neonatal calves (Malmuthuge et al., 2019). In fact, fresh colostrum reduced DNA copies of enteropathogens (*C. perfringes*, *E. coli*) in 7-day-old calves (Martin et al., 2021). Moreover, extended colostrum feeding for 3 days correlated with an abundance of mucosa-attached *Lactobacillus* spp. and *E. coli* in colons (Hromadkova et al., 2020). Our results support further studies to determine roles of colostrum to safeguard a healthy microbiome during enteric diseases in neonatal calves.

In summary, we described the damaging impact of *C. parvum* infection in neonatal calves, provoking severe diarrheic neutrophilic enterocolitis, perhaps augmented due to the lack of fully developed innate gut defenses at this young age. Colostrum supplementation (once per day after *C. parvum* challenge) showed limited effect mitigating diarrhea but had a positive effect on the associated, depression and required treatments in calves, as well as specific modulatory influence on host gut immune responses. Other colostrum formulations or regimes (e.g., doses and times) should be studied, exploring the abilities to regulate intestinal wellness further. For instance, cryptosporidiosis models with a lower infecting challenge could more accurately represent natural infections, given the marked susceptibility of newborn calves. Additionally, the use of colostrum beyond the first week of infection may better document the long-term impact of this management strategy on cryptosporidiosis diarrhea and associated clinical signs. Understanding of gut inflammatory and anti-parasite mechanisms during cryptosporidiosis and the testing of therapeutic alternatives remains critical due to the high mortality, severe outcomes, and zoonotic risk of *C. parvum.*

## Data availability statement

The proteomic raw data is available in the ProteomeXchange database under project accession: PXD040269.

## Ethics statement

The animal study was reviewed and approved by Canadian Guidelines for Animal Welfare (CGAW) and the University of Calgary Animal Care Committee (AC19-0051).

## Author contributions

LG, SS, MC, and CW conducted the experiments in calves and acquired the clinical data. PL, DY, and AD contributed the proteomic assessment and analysis. KC and AH performed the histology and staining assessment and qPCR studies. PS, SO, and DG performed the microbiome analysis. LG, CW, and EC participated in conceiving the experiments and designed the experiments. LG, KC, and EC wrote the manuscript and prepared the figures. All authors contributed to the article and approved the submitted version.
