# Supplementary material for: Host innate immune responses and microbiome profile of neonatal calves challenged with Cryptosporidium parvum and the effect of bovine colostrum supplementation
Source: Front Cell Infect Microbiol. 2023 May 3;13:1165312. doi: 10.3389/fcimb.2023.1165312 (PMC10189047; doi:10.3389/fcimb.2023.1165312)
Supplement: Supplementary file 1 [file DataSheet_1.pdf]

SUPPLEMENTARY MATERIAL

Gamsjäger et al.,

Host innate immune responses and microbiome profile of neonatal calves challenged with *Cryptosporidium parvum* and the effect of prolonged bovine colostrum supplementation

Supplementary Table 1. Shotgun proteomic proteins recognized in ilea from calves challenged by *Cryptosporidium parvum* (*C. parvum*) and supplemented with colostrum (C) or milk replacer (MR) (groups Sham/MR, Sham/C, *C. parvum*/MR, and *C. parvum*/C). Proteins were identified without constraint by enzyme specificity rules during spectrum-to-sequence matching. Proteins were identified from the bovine UniProt protein database using the Andromeda algorithm as implemented in the MaxQuant software (v.1.6.0.1) using a peptide FDR of 0.01. The bovine proteome was complemented by common contamination protein entries and reverse decoy sequences. Search parameters included a mass tolerance of 20 p.p.m. for the parent ion, 0.5 Da for the fragment ion, carbamidomethylation of cysteine residues (+57.021464 Da), variable N-terminal modification by acetylation (+42.010565 Da), and variable methionine oxidation (+15.994915 Da). TMT 6-plex labels 126 to 131 were defined as labels for relative quantification. The cleavage site specificity was set to Trypsin/P (search for free N-terminus and for only lysine), with up to two missed cleavages allowed.

Supplementary Table 2. Selected proteins in ilea challenged by *Cryptosporidium parvum* (*C. parvum*) and supplemented with colostrum (C) or milk replacer (MR) that were identified by

proteomics as upregulated and downregulated pathways upon comparisons. These includes Sham/MR: Sham/C, *C. parvum*/C: *C. parvum*/MR, *C. parvum*/MR vs Sham/MR, *C. parvum*/C vs Sham/C, and differentially regulated *C. parvum*-specific proteins in *C. parvum*/C: *C.parvum*/MR ilea. Data were analyzed using MaxQuant at a 1% false discovery rate (FDR), and data integration for pathway and gene ontology (GO) enrichment was performed with Metascape. Changes are expressed as abundance of proteins by $\log_2$, in which means $\log_2$ values >0 represent proteins that were upregulated (red) and $\log_2$ values <0 represent downregulated proteins (blue).

Supplementary Table 3. Virulence features identified in ilea challenged by *Cryptosporidium parvum* (*C. parvum*) and supplemented with colostrum (C) or milk replacer (MR).

Supplementary Figure 1. Transcription gene expression of pro-inflammatory cytokines and cathelicidin in ilea and colons of newborn calves orally challenged by *C. parvum* and supplemented with milk replacer or colostrum. mRNA expression of *TNFα*, *IFNγ*, *IL10*, and *cathelicidin 5* in (**A**) ilea and (**B**) colons from Sham and *C. parvum* (5 x10^7 oocysts) challenged calves fed with colostrum (Sham/C and *C. parvum/*C, respectively) or milk replacer (Sham/MR and *C. parvum/*MR, respectively) and terminated at 6 dpc. Data was determined by qPCR and represented relative to GAPDH. Data are means ± SEM. $P < 0.05$ (one-way ANOVA post hoc Bonferroni correction for multiple group comparison or two-tailed Student's t-test for 2 groups) was considered significant.

Supplementary Figure 2. Protein-protein interaction in ilea of calves orally challenged by *C. parvum* and supplemented with milk replacer or colostrum. STRING analysis of proteins in ilea (**A**) downregulated in Sham calves supplemented with colostrum (Sham/C vs Sham/MR), (**B**) upregulated in *C. parvum* challenged and supplemented with milk replacer (*C. parvum/*MR vs *C. parvum/*C), and (**C**) downregulated in *C. parvum* challenged and supplemented with milk replacer (*C. parvum/*MR vs Sham/MR). Ilea were collected at 6 dpc.

Supplementary Figure 3. Principal component analysis (PCA) interaction in ilea of calves orally challenged by *C. parvum* and supplemented with milk replacer or colostrum. Microbiota information collected from feces at 0 (reverse triangle) and 6 dpc (squares) and color coded according to groups (feeding regimen * infection status) as depicted in **Fig 7A**. Same abundance matrix used for the NMDS in **Fig 7A** was used after imputing 0s and CLR-transformation.

Supplementary Figure 4. Virulence features in ilea of calves orally challenged by *C. parvum* and supplemented with milk replacer or colostrum. A differential representation analysis in fecal microbiota at 6 dpc separated by the infection status. The sharpest contrasted features between groups (indicspecies stat >0.7, $P < 0.05$) are illustrated. Scatterplots show the abundance of the features in each sample.
