# Supplementary figures and images for: Host innate immune responses and microbiome profile of neonatal calves challenged with Cryptosporidium parvum and the effect of bovine colostrum supplementation
Source: Front Cell Infect Microbiol. 2023 May 3;13:1165312. doi: 10.3389/fcimb.2023.1165312 (PMC10189047; doi:10.3389/fcimb.2023.1165312)

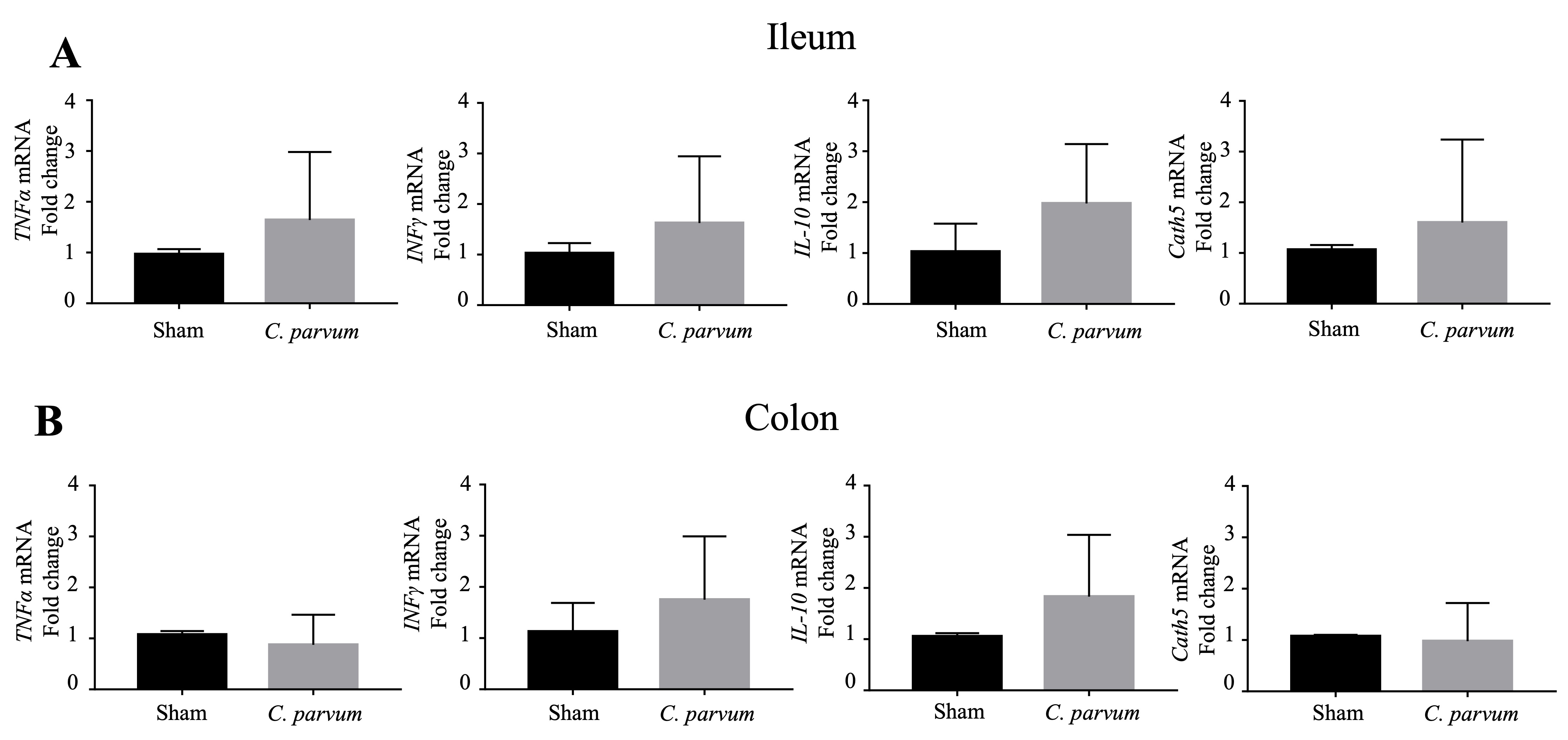

Supplement: Supplementary file 2 [file Image_1.jpeg]

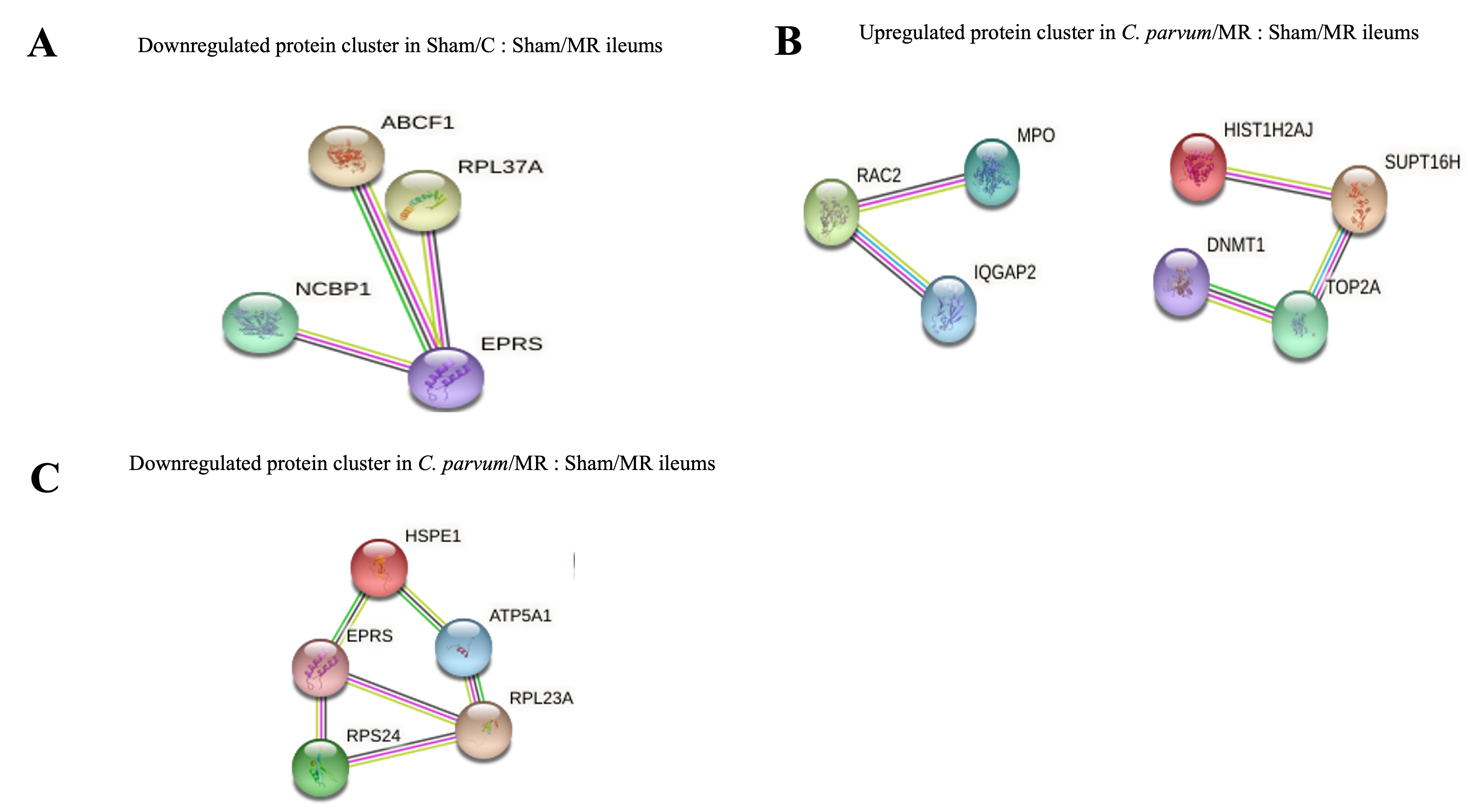

Supplement: Supplementary file 3 [file Image_2.jpeg]

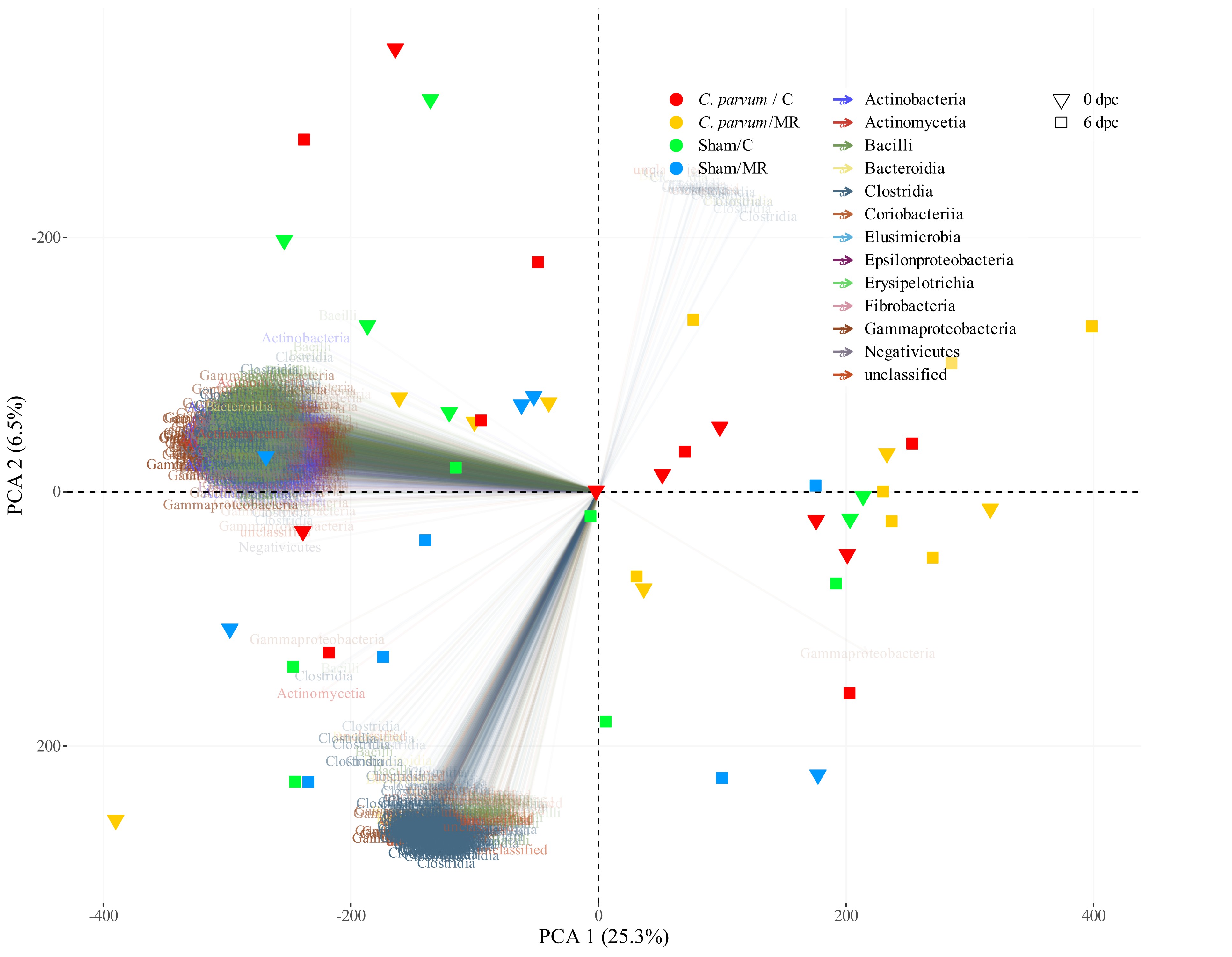

Supplement: Supplementary file 4 [file Image_3.jpeg]

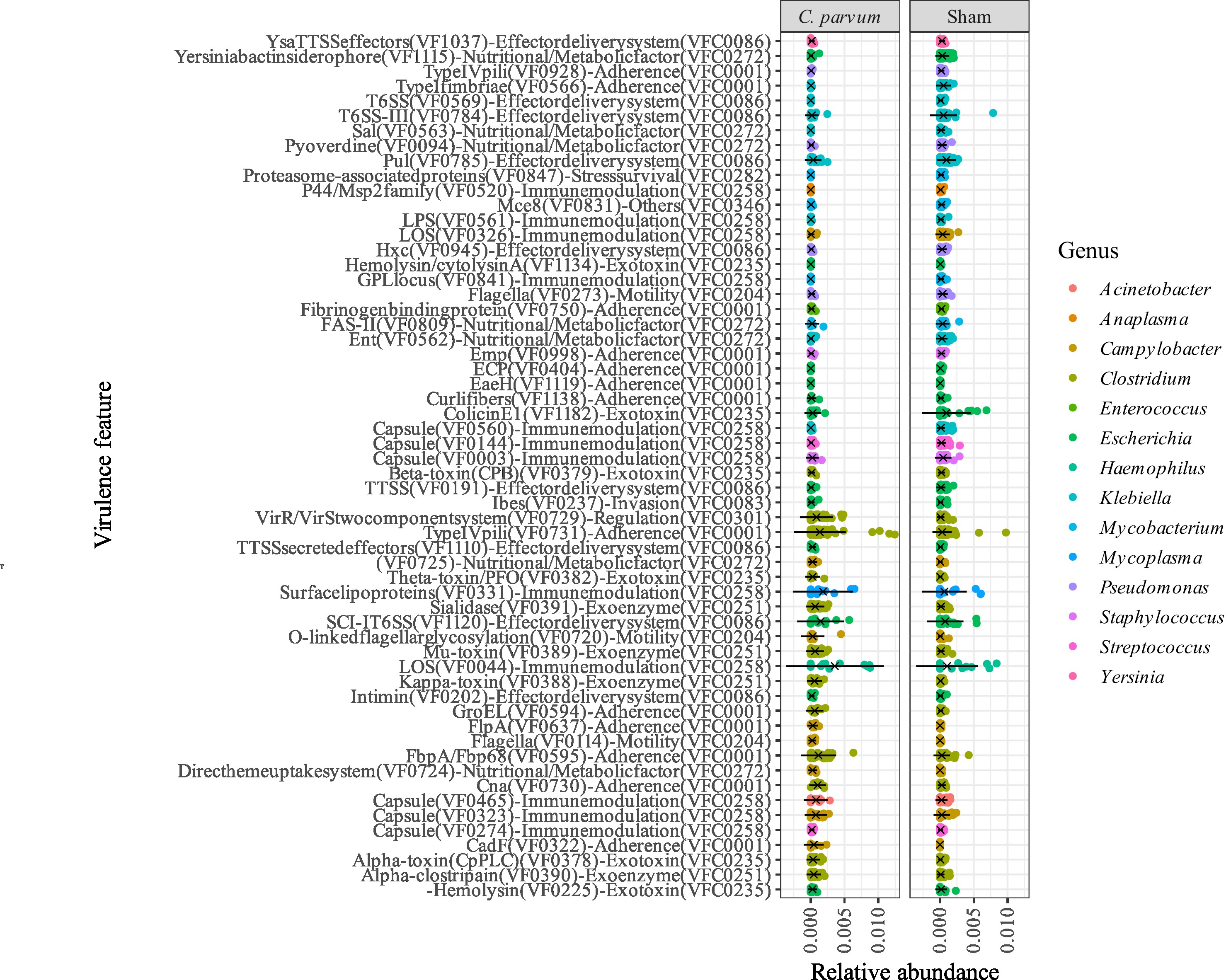

Supplement: Supplementary file 5 [file Image_4.jpeg]
